# Oxalate secretion by ectomycorrhizal *Paxillus involutus* is mineral-specific and controls calcium weathering from minerals

**DOI:** 10.1038/srep12187

**Published:** 2015-07-22

**Authors:** A. Schmalenberger, A. L. Duran, A. W. Bray, J. Bridge, S. Bonneville, L. G. Benning, M. E. Romero-Gonzalez, J. R. Leake, S. A. Banwart

**Affiliations:** 1 Cell-Mineral Research Centre, Kroto Research Institute, University of Sheffield, S3 7HQ, UK; 2 Animal and Plant Sciences, University of Sheffield, S10 2TN, UK; 3 Life Sciences, University of Limerick, Limerick, Ireland; 4 Earth Surface Science Institute, School of Earth and Environment, University of Leeds, Leeds LS2 9JT, UK; 5 GFZ, German Research Centre for Geosciences, Telegrafenberg, Potsdam 14473, Germany

## Abstract

Trees and their associated rhizosphere organisms play a major role in mineral weathering driving calcium fluxes from the continents to the oceans that ultimately control long-term atmospheric CO_2_ and climate through the geochemical carbon cycle. Photosynthate allocation to tree roots and their mycorrhizal fungi is hypothesized to fuel the active secretion of protons and organic chelators that enhance calcium dissolution at fungal-mineral interfaces. This was tested using ^14^CO_2_ supplied to shoots of *Pinus sylvestris* ectomycorrhizal with the widespread fungus *Paxillus involutus* in monoxenic microcosms, revealing preferential allocation by the fungus of plant photoassimilate to weather grains of limestone and silicates each with a combined calcium and magnesium content of over 10 wt.%. Hyphae had acidic surfaces and linear accumulation of weathered calcium with secreted oxalate, increasing significantly in sequence: quartz, granite < basalt, olivine, limestone < gabbro. These findings confirmed the role of mineral-specific oxalate exudation in ectomycorrhizal weathering to dissolve calcium bearing minerals, thus contributing to the geochemical carbon cycle.

The formation of soil, which provides the foundations of terrestrial ecosystems and agriculture, is based on the weathering of rock-forming minerals^1^. Calcium and magnesium are abundant in the Earth’s crust^2^ where calcium in particular precipitates with the bulk of the Earth’s orthophosphate. Biological acquisition of growth-limiting mineral phosphorus is therefore expected to mobilise calcium in excess of biological requirements through biologically-driven co-dissolution from phosphorus-bearing minerals. Quantification of weathering activities, particularly for calcium- and magnesium-bearing rocks and minerals, is a key component in predicting rates of soil formation, calcium and magnesium fluxes from the continents to the oceans, and fluxes of biologically essential elements including phosphorus. The release of such key nutrients ultimately control the biomass and productivity in long-term ecosystem developments^3^,^4^,^5^.

Until recently, continental-scale biological weathering has been largely attributed to plant root activities^6^,^7^. However, 80–90% of all plant species associate with mycorrhizal fungal partners^8^,^9^. Mycorrhizal mycelial networks are several times longer and much finer than plant roots, in the case of ectomycorrhiza often extending to 200 m in length g^−1^ soil^10^. These networks are supported by up to 30% of the net photosynthate of their plant hosts^10^. In return, the mycelial networks enhance plant uptake of growth-limiting nutrients, in particular phosphorus, from the soil environment thereby enhancing ecosystem productivity^11^,^12^,^13^. In typical ectomycorrhizal forests, which comprise over 35% of the global forest cover^14^, the transfer of photosynthate from tree-to-mycorrhizal fungi amounts to a biotic energy flux of 1000–6000 kJ m^−2^ a^−1^, (based on solar energy capture of average insolation for temperate forests)^15^. This substantial energy input from plants into mycorrhiza is not only used for catabolic reactions (e.g. respiration) by the fungi, but also allocated to nutrient acquisition from minerals through, amongst other processes, the exudation of organic acids^8^,^16^ that accelerate chemical dissolution of oxide and silicate minerals. Despite increasing awareness of the potential importance of these plant-fungal partnerships in mineral weathering, a paucity of measurements has quantified their contributions to element mass transfers. Likewise, the extent to which plant photosynthate is directed to fungal-mineral interfaces has received limited attention to date^17^, but has recently been shown to be a major control on mycorrhizal-fungal driven mineral weathering rates^18^.

A number of ectomycorrhizal fungi, including *Paxillus involutus*, can be grown in pure culture, where the fungi can synthesise and secrete up to 1 mmol oxalate g^−1^ of fungal biomass (DW)^19^,^20^,^21^. Both calcium and bicarbonate ion concentrations influence oxalic acid secretion by *P. involutus* grown with nitrate^19^,^20^,^21^. The secretion of oxalic acid is thought to play a major role in the dissolution of apatite, releasing phosphorus for uptake by ectomycorrhizal fungi and immobilizing the released calcium into calcium oxalate^22^. However, other elements may also influence fungal oxalate secretion. For example, *Pinus sylvestris* symbiotic with *Hebeloma crustuliniforme* has shown trends for oxalate exudation to be higher when exposed to rock grains containing different amounts of potassium^23^. This is consistent with studies of fungi in ectomycorrhizal symbiosis with trees acting as biosensors actively foraging for nutrients in rocks and minerals, distinguishing mineralogy such as apatite from quartz, and different grain sizes^17^,^24^,^25^.

In the current literature, most mineral weathering data are derived from solution-based abiotic dissolution experiments^26^,^27^,^28^. There, weathering of rocks and minerals is controlled primarily by interactions in aqueous solutions^29^ in which rates are dependent on the distance from equilibrium, pH, ionic strength, redox state, and temperature. Biological weathering processes are distinct in their spatial precision, often targeted at specific nutrient-rich minerals in soils, evident as preferential colonization of mycorrhizal hyphae on grains of certain rock types^24^. Some of the most biologically active forest soils that experience intense weathering from organic acid exudation by mycorrhizal fungi are freely-draining and seasonally dry^30^. Consequently, there is a need to study weathering in environments where soils are not water saturated for extended periods of time^31^, e.g. in the absence of gravitational water^32^. Indeed, weathering of biotite and apatite in microcosms by *P. involutus* in symbiosis with host trees has revealed that significant chemical and physical alteration of minerals can take place in conditions where the minerals are not immersed in water^22^,^33^,^34^,^35^.

The aim of the present study was to test the hypotheses that (i) ectomycorrhizal weathering is ultimately driven by fungal allocation of photoassimilate received from a host plant (in this case, *Pinus sylvestris*), (ii) the release of calcium from minerals, and its accumulation as a weathering product by a symbiotic fungal partner (in this case, *P. involutus*), is linked to mineral-specific rates of fungal secretion of oxalic acid and the formation of calcium oxalate. To address these hyphotheses, monoxenic microcosms were designed with mineral weathering arenas, containing grains of a range of important carbonate and silicate rocks and minerals (limestone, basalt, gabbro, granite, olivine, microcline) of various calcium and phosphorus concentrations and a control mineral (quartz, 0.01% CaO, 0.0006% P_2_O_5_). These rocks/minerals were accessible only to mycorrhizal fungal hyphae extending from the roots of the host plants^17^ and were hydrologically isolated from the root/mycorrhiza compartments to mimic unsaturated soil environments in which ectomycorrhizal fungi are most active^9^. The allocation of recent photosynthate carbon to *P. involutus*, and its subsequent deployment in weathering arenas was quantified using ^14^CO_2_ pulse-labelling and tracing. Quantification of calcium oxalate accumulation on symbiotic ectomycorrhizal fungal hyphae revealed the extent to which calcium mass transfer, resulting from plant-drived fungal weathering is linked to oxalate secretion.

## Results

### Rock/mineral composition

Concentrations in the rocks and minerals were expressed as weight (wt. %) binary oxide (Table 1). Calcium oxide (CaO) ranged from 32% in the limestone, to 15% in gabbro, 11% in basalt, 4% in granite and less than 0.2% in olivine and microcline. For magnesium oxide, (MgO) the highest concentrations were found in olivine (45%), followed by limestone (14%), gabbro (10%) and basalt (8%). In contrast, the highest amounts of potassium oxide (K_2_O) were identified in microcline (15%) and granite (4%). Phosphorus concentrations were low in all rocks/minerals (0.004–0.36% P_2_O_5_) but varied by almost two orders of magnitude (Table 1) around the average concentration of phosphorus in the earth’s crust^2^. The bulk chemical compositions of the rock/mineral samples are reflected in the mineralogy, as determined by Rietveld refinement of XRD patterns (Table 2). Samples of granite contained anorthite (16%, CaAl_2_Si_2_O_8_) as the only calcium bearing mineral. Basalt samples contained anorthite (59%), and augite (20%, (Ca,Na)(Mg,Fe,Al)(Si,Al)_2_O_6_), while samples of gabbro contained anorthite (56%) and diopside (13%, CaMgSi_2_O_6_) as the major calcium bearing minerals. Limestone was primarily comprised of dolomite (74%, CaMg(CO_3_)_2_).

### Plant-to-fungal carbon allocation to weathering arenas

**Figure 1:**
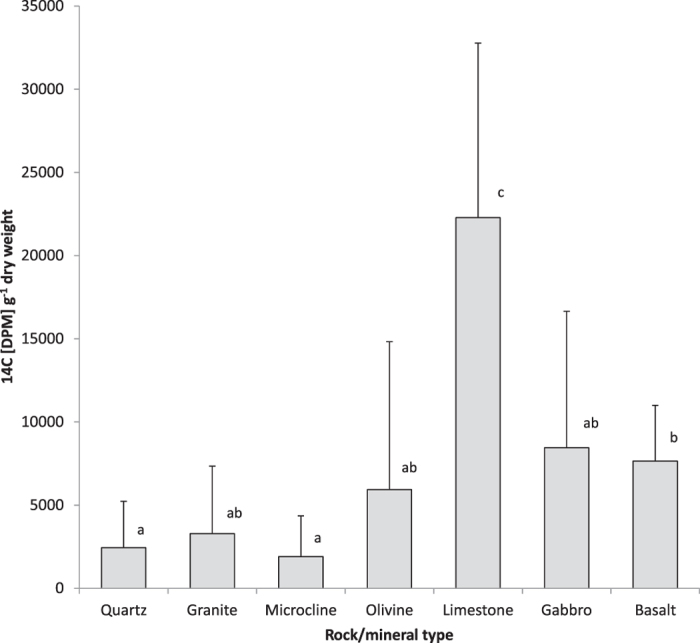
Quantification of ^14^C pulse-labelled photosynthate allocated by *Pinus sylvestris* to *P. involutus*, and its subsequent deployment in biomass and exudates in fungal-colonised weathering arenas containing quartz, granite, microcline, basalt, olivine, limestone and gabbro. Error bars indicate standard deviation. Means sharing the same letter are not significantly different (P > 0.05, ANOVA, Games-Howell).

Amounts of ^14^C fixed by *Pinus sylvestris* and allocated from roots to weathering arenas colonised by *P. involutus* were lowest in wells with quartz, granite and microcline with below 3300 disintegrations per minute (DPM) g^−1^ dry weight (DW) (Fig. 1). A higher ^14^C allocation of over 5900 DPM g^−1^ DW was recorded in the wells with olivine although this difference was not significant (P > 0.05). ^14^C allocations for colonised wells with basalt and gabbro were close to 8000 DPM g^−1^ DW and for basalt significantly higher than quartz and microcline (P < 0.05). The highest ^14^C carbon allocations of over 22,000 DPM g^−1^ DW were detected in colonised wells with limestone (P < 0.05).

### Determination of pH of *P. involutus* hyphae in weathering arenas

**Figure 2:**
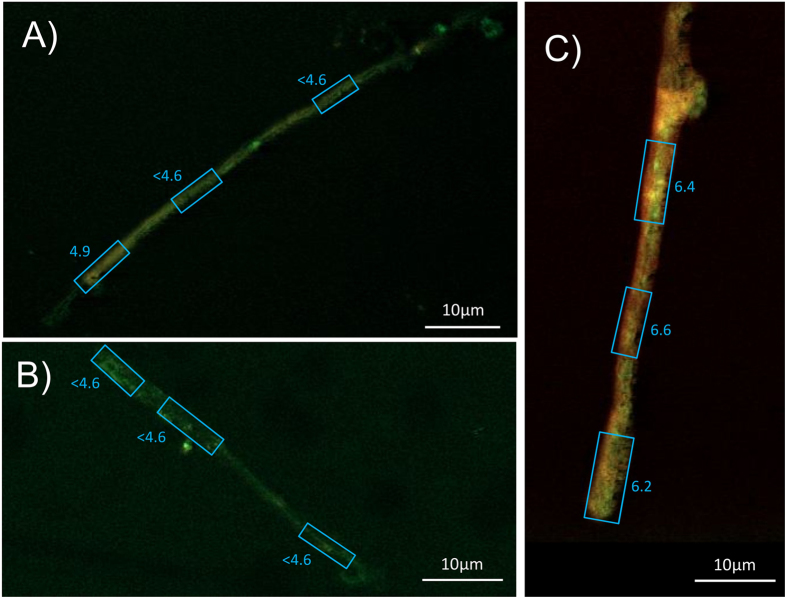
Confocal laser scanning microcscope images from symbiotic *P. involutus* hyphae stained with 5 μM SNARF4F (Invitrogen) in water and emmissions detected at 580 and 640 nm with a Zeiss Axioplan and a 510 meta detector, excited with a 488 nm argon laser (see materials for details). Fluorescence intensities revealed pH values for (**A**) *P. involutus* from basalt, pH < 4.6–4.9, (**B**) *P. involutus* from limestone, pH < 4.6, (**C**) *P. involutus* from granite, pH = 6.2–6.6.

The pH values of the mycorrhizal hyphae removed from the grains of granite, basalt and limestone were determined with the fluorescent molecular probe SNARF4F through confocal laser scanning microscopy (CLSM). While the probe signal on the hyphae taken from wells with limestone and basalt resulted in preferential emission of fluorescent light around 580 nm (green), fungal samples taken from the granite wells typically showed stronger emissions at 640 nm than at 580 nm. Calibration of the signals revealed a pH from 4.9 to below 4.6 (pH 4.6 was the lowest calibration point, Fig. 2a,b) for basalt and limestone derived samples. In contrast, hyphae taken from the granite wells had variations in the fluorescence signal in the range of pH 6.2 to 6.6 (Fig. 2c).

### Qualitative analysis of the chemistry of symbiotic hyphae and attached secondary minerals from weathering arenas through micro Fourier transform infrared (*μ*FT-IR) spectroscopic analysis

**Figure 3:**
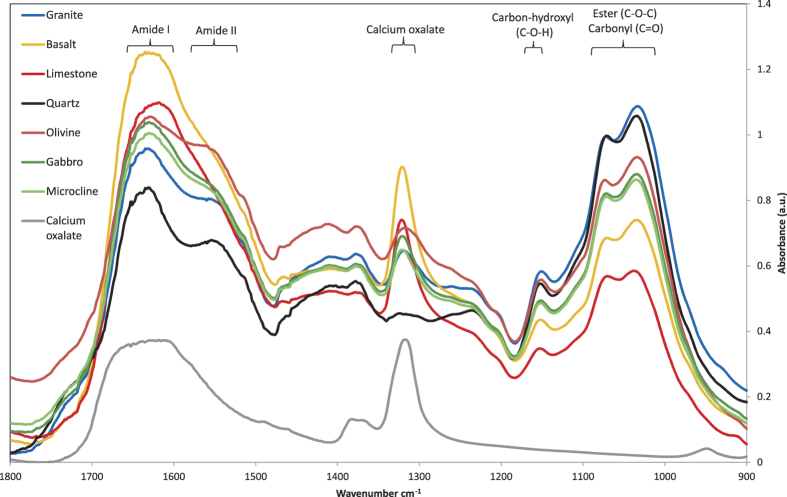
Micro Fourier transform infrared (*μ*FT-IR) spectra from symbiotically grown hyphae of *P. involutus* with adhering secondary minerals taken from weathering arenas in microcosms with minerals/rocks of quartz (black), granite (blue), microcline (bright green), basalt (orange), olivine (dark red), limestone (bright red) and gabbro (dark green). Calcium oxalate was measured in comparison as a fine powder (grey). Values were obtained from combined readings from 6 different locations of each sample in triplicates at 100 repeats each (1800 repeats per spectrum in graph).

All sampled *P. involutus* hyphae removed from microcosms with grains of olivine, basalt, limestone and gabbro showed a characteristic peak at 1317–1324 wavenumber cm^−1^ in the *μ*FT-IR spectrum, identical to the main peak for calcium oxalate monohydrate^36^ (Fig. 3). The fungal amide peaks I and II were overlaid with a broader second calcium oxalate peak at approximately 1628 cm^−1^ with the result that the amide II peak (1550 cm^−1^)^37^ was only detectable as an inflexion point (as a result of putative calcium oxalate accumulation) from the 1628 cm^−1^ maximum. A characteristic peak of calcium oxalate between 1317–1322 cm^−1^, and a partial overlay of the amide peaks at 1628 cm^−1^ were detectable, associated with some hyphal samples removed from two of six wells with granite and microcline while all hyphal samples removed from wells with quartz grains showed no presence of calcium oxalate. Three additional characteristic peaks were detected in all fungal samples in the region of 1030–1160 cm^−1^ referred to here as carbon-hydroxyls, esters and carbonyl groups. This region has also been referred to as polysaccharides of fungal mycelia^37^.

### Qualitative analysis of crystals formed on hyphae employing scanning electron microscopy with energy dispersive X-ray spectroscopy (SEM-EDS)

*P. involutus* hyphae were taken from a well in representative microcosms with limestone, basalt and granite grains respectively and analysed using SEM-EDS. The electron backscatter images of all the fungal hyphae from wells with limestone and basalt showed bright crystalline structures, while only a few of these particles were found associated with hyphae removed from a microcosm with granite grains (Supplementary Fig. S1). EDS analysis of selected areas with those particles at the surface of mineral grains confirmed the presence of calcium. EDS from hyphal samples from basalt and limestone wells showed peaks at K_α_ (emission line of electron transition to K shell) of calcium that reached about 3000 and 6000 X-ray counts per minute, while comparatively smaller peaks from selected areas of hyphal samples from granite only reached levels of less than 800 X-ray counts per minute.

### Quantitative analysis of oxalate and calcium in and attached to the surface of symbiotic hyphae using ion chromatography and inductively coupled plasma mass spectrometry

Amounts of oxalate per unit biomass of hyphae increased in order from quartz, granite, microcline, basalt, olivine, limestone to gabbro (Table 3). Hyphae of *P. involutus* sampled from wells with quartz and granite contained less than 10 mg oxalate per g of biomass (DW, including secondary minerals, Table 3). Significantly higher (P < 0.05) concentrations of oxalate were found in the presence of basalt, olivine and limestone in the range of 60–80 mg oxalate g^−1^ biomass (DW, including secondary minerals). Over 190 mg oxalate g^−1^ biomass (DW, including secondary minerals) was found in the presence of gabbro, which was higher (P < 0.05) than for all other minerals or rocks tested.

While very low concentrations of calcium were detected associated with fungal biomass growing with quartz and granite (below 4 mg g^−1^ biomass DW, including secondary minerals), higher values were found with hyphae growing on microcline and olivine (20–40 mg g^−1^ biomass DW including secondary minerals). Hyphae in the presence of basalt, limestone and gabbro (>10% CaO in parental rocks, Table 1) showed significantly higher (P < 0.05) accumulation of calcium (40–120 mg g^−1^ biomass DW including secondary minerals) with gabbro providing significantly higher values than all other tested minerals and rocks (P < 0.05; Table 3). In contrast, hyphae of *P. involutus* grown in monoculture on Modified Melin Norkran’s agar contained only 1.4 mg g^−1^ biomass DW calcium. Accumulation of oxalate and calcium per unit length of hyphae per day showed close similarities to the total accumulation, with hyphae from weathering arenas of gabbro exceeding the accumulation of 2.1 and 2.7 10^−10^ mol m^−1^ d^−1^ oxalate and calcium, respectively (Table 4).

**Figure 4:**
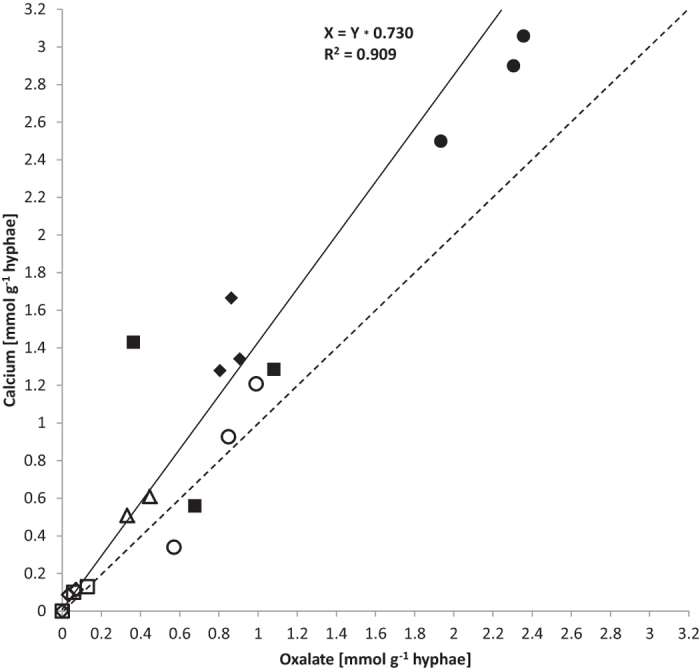
Correlation of oxalate and calcium accumulation rates in mmol g^−1^ DW from quartz (empty diamond), granite (empty square), microcline (empty triangle), olivine (empty circle), basalt (filled square), limestone (filled diamond) and gabbro (filled circle). R-square value of linear regression fit is 0.909, at a slope of 1.37 (black line) in comparison to a slope of 1 (dashed line).

Calcium and oxalate accumulation per g biomass (DW, Table 3) correlated closely (calcium:oxalate, R^2^ = 0.909) at a slope of 1.37 (Fig. 4). For olivine and granite the mass ratio of calcium:oxalate was closer to 1.0 while for limestone and basalt it was closer to 1.4. The noble agar base in the wells was a potential source of calcium. However, concentrations were only in the region of 11.5 (+/−0.02) ng g^−1^ (wet weight, WW). Outside the wells, the base agar concentrations ranged around 16–19 ng g^−1^ WW for calcium that was separated from the fungus by a cellophane sheet. The perlite was a further potential source of calcium (Table 1).

### Mycorrhizal weathering rates compared to abiotic weathering rates

This study’s fungal-driven calcium accumulation rates (moles Ca m^−2^ s^−1^) were based on the maximum theoretical extent of hypha-mineral contact area as the mass transfer interface and represent lower bound values as; i) large proportions of the hyphae have been observed to be not in direct contact with rock/minearls, thus the maximal fungal-mineral contact area may be greatly overestimated and as a consequence the calculated calcium accumulation rate per square metre underestimated, possibly by a factor of 10 based on visual inspection of microcosms and optical microscopy micrographs; ii) duration of colonization of weathering arenas may have been up to two weeks shorter than reported due to biweekly colonization inspections; iii) fungal oxalate secretion may not have taken place at a constant rate during the colonization of the weathering arenas; iv) not all calcium and oxalate may have been transformed into secondary minerals, for example, some calcium may be transported to the plant host, and not included in this quantification.

Accumulation rates of calcium through fungal-mineral interaction in monoxenic microcosms in this study were greater than reported abiotic stoichiometric weathering rates of pure calcium bearing mineral phases in laboratory experiments at the same temperature^26^,^28^,^38^. Based on the presence of anorthite, the fastest weathering calcium bearing mineral in basalt and gabbro, previously reported abiotic weathering rates at pH 4 were 10–100 times lower than the here quantified ectomycorrhiza weathering rates (even without considering the factors discussed above that are likely to have underestimated this biotic weathering). However, calcium accumulation rates from granite in this study were similar to the abiotic dissolution rates reported for anorthite. Furthermore, calcium accumulation from limestone weathering in this study was at least three orders of magnitude lower than reported abiotic dissolution of dolomite^38^ (Table 5a,b).

## Discussion

The formation of secondary minerals on hyphae in this study was confirmed to be calcium oxalate using *μ*FT-IR^39^ and these findings were verified via identification of calcium as one main component of the crystals using SEM-EDS. The overlay of the calcium oxalate spectrum and the amide I and II peaks of the fungal biomass allowed a semiquantitative evaluation suggesting that exudation of higher quantities of oxalate were correlated to rock/mineral samples containing more than 10% wt of calcium and magnesium combined (olivine, basalt, gabbro, and limestone). No other types of oxalate crystals such as magnesium oxalate (glushinskite) were identified via *μ*FT-IR. Likewise, SEM-EDS identfied no major cations other than calcium in the crystals. Minor variations in the IR peak position of calcium oxalate were most likely associated with crystaline water (in mono- and dihydrate forms)^40^. The lack of accumulation of other oxalate based crystals such as magnesium oxalate suggested either calcium-selective dissolution or that nutrients other than calcium such as magnesium may have dissolved and been taken up by the fungal hyphae and transported to the plant host.

In this study, the pH at the hyphal-surfaces was lowered from above 6 to below 5 in weathering arenas with basalt and limestone when compared to granite. By analogy with abiotic dissolution mechanisms, this drop in pH, putativley associated to the potential uptake of cations by the hyphae and the exudation of oxalate (~10^−10^ mol m^−1^ d^−1^; Table 4), would accelerate calcium-bearing silicate and carbonate mineral dissolution that includes the release of calcium and production of calcium oxalate on the fungal hyphae as identified via *μ*FT-IR. These findings are in accord with previous observations of symbiotic *P. involutus* hyphae on biotite, where pH decrease and organic acid exudation, amongst other processes, was identified as an important factor in fungal directed weathering^33^. The lower pH values for hypha in contact with basalt and limestone are associated with relatively greater calcium release, compared with higher pH values for hypha in contact with granite which exhibited far less calcium release (Fig. 2). Because the associated alkalinity production from calcium dissolution should increase interfacial pH, this indicates that the relatively lower interface pH in the presence of the greater calcium dissolution for basalt and limestone is controlled independently by biologicaly-induced mechanisms such as cation uptake and oxalate production resulting in calcium oxalate precipitation, rather than as a consequence of the abiotic calcium dissolution reaction. A biologically-driven regulation of interface pH is also supported by the correlating measurements of greater ^14^C photosynthate allocation to basalt and limestone compared to granite (Fig. 1).

The fungal allocation of ^14^C-labelled carbon received from tree photosynthesis differed between groups of rocks and minerals, with low allocations to granite, microcline and quartz, medium-high allocations to basalt, gabbro and olivine and significantly higher allocation to limestone grains (Fig. 1). The accumulation of calcium on the hyphae correlated with ^14^C allocation (R^2^ = 0.688 for silicate rocks and minerals; Supplementary Figure S2), with low amounts associated with hyphae in the presence of granite and quartz, and significantly higher amounts of calcium were associated with hyphae taken from arenas with basalt, limestone and gabbro (Table 3). Likewise, accumulation of oxalate was lowest in/on hyphae in the presence of granite and quartz, and significantly higher in/on hyphae taken from arenas with olivine, basalt, limestone and gabbro. This is consistent with a previous study^22^, employing almost identical microcosms with *P. sylvestris* symbiotic with *P. involutus*, in which smallest amounts of ^14^C-labelled recent photosynthate were traced into fungal-colonised weathering arenas containing quartz compared to much higher rates of ^14^C allocation into those containing apatite grains.

Consequently, the evidence suggests that the allocation of photosynthate in ectomycorrhizal *P. involutus* mycelial networks is intimately linked to factors that enhance the dissolution reactivity of calcium-bearing silicate and carbonate minerals. These include; active lowering of pH, increased exudation of oxalate as a ligand to accelerate element release from mineral surfaces, resulting in calcium oxalate precipitation. This reduces the dissolved calcium activity thereby maintaining high solid-solution concentration gradients favouring dissolution of calcium-bearing silicate and carbonate minerals. These factors all correlate with the observed biological effects of increased photoassimilate allocation, oxalate exudation and the release of elements from minerals. However, this link was weaker in weathering arenas with limestone, in which ^14^C accumulation was higher than for other minerals but the accumulations of oxalate-bound calcium was significantly less than in the presence of gabbro. This may be due to some of the ^14^C in the case of the limestone arising from abiotic precipitation / exchange of ^14^C into the calcium carbonate mineral.

The stoichiometry of calcium oxalate predicts a one-to-one ratio for the crystalline weathering product, whereas in this study ratios (calcium:oxalate) across the arenas with different minerals and rocks were somewhat higher at 1.37 (R^2^ = 0.909; Fig. 4, Table 3). Calcium abundance exceeding that of oxalate may have come in part from calcium in the fungal biomass which would not be part of the secondary minerals.

While only in the weathering arena with gabbro the fungal biomass was singificantly higher than in the quartz controls, recent investigations of rock grains buried under angiosperm trees also revealed significantly higher hyphal colonization rates (quantified as hyphal length) of basalt compared to granite and quartz^24^. Findings in the present study suggest that after initial exploratory colonization of weathering arenas, the presence of certain rocks/minerals have subsequently resulted in enhanced weathering activity, but not necessarily in significant increases in fungal biomass. However, photosynthate (^14^C) allocation was elevated in weathering arenas of higher weathering activity (calcium oxalate accumulation), albeit not always significantly. In this study, ^14^C allocation was measured at the end of the experiment over a period of 19 hours. The biological activity of the fungus in growth and secretion of oxalate during the 19 h of ^14^C exposure is likely to have changed very much from the initial colonization to the later stages of mineral dissolution throughout the experimental period. Some of the high variability in ^14^C allocation by the fungus among the measured replicates may reflect temporal differences in rates of colonization of the wells containing minerals.

The present study revealed that the accumulation of oxalate, not the accumulation of fungal biomass (P = 0.217), served as a proxy of fungal weathering activity by symbiotic *P. involutus* and was linked to the photoassimilate allocation at the weathering interface. It is unlikely that biological regulation of weathering activity was solely based on calcium release, since calcium requirements by the plant host (10 to 50 mg g^−1^ (DW) in plant tissue)^41^ are rather limited. The mobilization of other nutrients, essential for growth are likely to have played a key role. Only traces of essential nutrients such as phosphorus, magnesium or potassium are present in quartz, thus photosynthate allocation to quartz by the fungus would be uneconomical as opposed to more nutrient-rich rocks or minerals. Olivine, limestone, gabbro and basalt contained high levels of magnesium, but in different mineral forms (forsterite, dolomite, diopside and augite, respectively). Basalt and granite contained the highest amounts of phosphorus. However, XRD analysis was unable to determine its mineral form. Since about 95% of phosphorus in Earth’s crust is associated with calcium and this element controls P solubility in most soils^42^, the calcium release by mycorrhiza may be a constitutive response to facilitate phosphorus uptake by plants since this is one of the primary functions of mycorrhiza^9^,^12^.

Quirk and colleagues concluded in their work that mycorrhizal fungi concentrate their activity on weatherable nutrients^24^. Indeed, the abiotic kinetic reactivity of calcium bearing minerals alone could not explain the level of accumulated calcium oxalate in this study. The abiotic dissolution rate of calcium from dolomite, the dominating calcium bearing mineral in limestone, is about five orders of magnitude higher than from anorthite^26^,^28^ (at pH 4), which is highly abundant in basalt and gabbro (Table 2). However, biotic calcium dissolution in this study was significantly higher from gabbro than limestone. These findings suggest that ectomycorrhizal weathering is highly targetted and enhanced by calcium-bearing minerals but the rate of oxalate release by *P. involutus* is not controlled soley by the calcium concentration in a mineral or rock. The accumulation of calcium oxalate indicates rates of calcium dissolution that exceed biological demand by the fungi and the plants, and the relatively low solubility of calcium oxalate. High levels of calcium may actually be toxic to fungi. Wood rotting fungi have been reported to form caclium oxalate crystals as a form of detoxification^43^, thus calcium oxalate formation in this study may have reduced a possible negative effect of free calcium on fungal growth.

The weathering of calcium-bearing minerals is likely to have released other nutrient elements that may have been taken up by the fungal hyphae and transported to the plant host, and would therefore not be detected on the fungal hyphae. Indeed, Smits and colleagues found evidence of phosphorus translocation from apatite through *P. involutus* to the plant host *P. sylvestris*^22^ and calcium accumulation most likely as calcium oxalate crystals have been reported in mesh bags with apatite buried in forest soils^44^. However, the presence of apatite in the present study could only be inferred from normative calculations. In olivine, calcium and phosphorus concentrations are very low. Nevertheless, oxalate accumulation on fungal hyphae in contact with olivine was found to be significanlty higher than with quartz or granite. This suggests that nutrients such as magnesium, abundant in olivine, basalt, gabbro and limestone may have been targetted by the fungus as well. The accumulation rates of oxalate in this study (Table 4) were of the same order of magnitude as in pine microcosms with *Hebeloma crustuliniforme* as fungal symbiont^23^.

Calcium concentrations in the plant host are about an order of magnitude lower than its typical concentrations in the earth’s crust^2^. The relatively low plant calcium demands means that the rate of calcium oxalate accumulation on the fungal hyphae in our study can be used as a proxy for estimating ectomycorrhizal weathering rates. These weathering rates were highest for gabbro calculated at 1.1 × 10^−9^ mol m^−2^ s^−1^ calcium and exceeded independently reported abiotic weathering rates of the calcium bearing minerals in gabbro (anorthite, diopside, augite, hornblende) by a factor of 100 and exceeded abiotic weathering rates by a factor of 10 in the case of calcium bearing minerals in basalt and olivine (Table 5). Unlike weathering rates from this study, abiotic weathering rates taken from the literature were carried out with individual mineral components only, thus abiotic weathering rates of minerals in rocks as used in this study may not be exactly the same. Since the calculations behind the ectomycorrhizal weathering rates assumed that all fungal hyphae were in contact with the rocks or minerals, the actual fungal weathering rate was potentially much higher as microscopic observations suggested that the vast majority of fungal hyphae were actually aerial. Considering the given uncertaincies from above, the weathering rates reported here are considered to be lower bound values.

The extent of calcium oxalate acumulation on the ectomycorrhizal hyphae alongside photosynthate translocation to the fungal hyphae clearly show that ectomycorrhizal weathering is indeed ultimately driven by the carbon supply provided by the plant. However, calcium abundance in the silicate rocks, minerals and limestone tested appears not to be the sole driver for targetted fungal weathering via exudation of oxalate to mobilize nutrients. This study provides indirect evidence that plant and fungal nutrient element contents in minerals, such as magnesium or phosphorus may contribute to the specific allocation of fungal exudates and biological weathering activity, but the main chemical sink for secreted oxalate is the formation of crystaline calcium oxalate, irrespective of the chemistry of the minerals.

Despite the limitations of deducing weathering rates from the accumulation of calcium in secondary minerals, calculations presented in this study clearly indicate that ectomycorrhial weathering has the potential to be several orders of magnitude faster than abiotic weathering rates. However, interaction of ectomycorrhizal hyphae in soils with other microbes such as bacteria may increase or decrease biological weathering. Oxalate may serve as a carbon source for a group of soil bacteria^45^ while at the same time soil bacteria may enhance biological weathering through organic acid release^27^. Furthermore, ectomycorrhiza other than *P. involutus* may show different rates of weathering. Thus, further research is needed to quantify the effect of other microbiota in biological weathering in soils in the future. In conclusion, ectomycorrhizal weathering has the potential to dominate chemical dissolution rates in the vadose zone of temperate forests where low levels of gravitational water and high ectomycorrhizal activity occur. The evidence provided in this study will further support long-term carbon cycle models where ectomycorrhiza-weathering is increasingly recognized as a driver of land-to-ocean calcium fluxes, influencing the long-term concentration of CO_2_ in the atmosphere^46^,^47^. This has been shown to occur via non-linear feedbacks, where as atmospheric CO_2_ decreases through being sequestered into marine calcium and magnesium carbonates, rates of tree-mycorrhiza-driven weathering also decrease as a result of restricted photosynthate allocation with falling CO_2_^18^,^48^.

## Methods

### Rock and mineral substrates used for fungal weathering experiments

Samples of rocks (granite, basalt, limestone, gabbro) and minerals (quartz, microcline and olivine) were individually fractured using a jaw-crusher and further broken in an agate mortar before sieving to obtain a 0.5–1.0 mm size fraction. These rock/mineral grains were then washed ultrasonically in deionised water to remove fine particles, dried at 80 °C and subsequently sterilised by autoclaving.

The chemical composition of the crushed rock/mineral samples was determined by X-ray Fluorescence (XRF) analysis using an XRF spectrometer (PANalytical Axios Sequential system; School of Earth, Atmospheric and Environmental Science, University of Manchester, UK). The mineralogy of each crushed sample was quantified by X-ray diffraction (XRD) using a Bruker D8 Advance Diffractometer with a scan range between 2 to 75° 2*θ* and at a 0.01° step size. The relative percentages of the mineral phases were determined by Rietveld refinement using the TOPAS software (version 4.2, Bruker AXS).

### Monoxenic microcosms with plant host and symbiotic fungus

*Pinus sylvestris* seeds from the Forestry Commission, England were surface sterilized in two rounds of 30% hydrogen peroxide solution for 5 min each, rinsed in sterile distilled water and aseptically germinated over a period of 4 weeks on 1.5% plant agar as described elsewhere^49^. Germination took place in climate controlled plant growth chambers at 15 °C day and 10 °C night temperatures, with an 18 hour photoperiod at a photon flux density of 250 μmol m^−2^ s^−1^ and relative humidity of 60–75%. These growth conditions were maintained throughout the experimental stages of the study. The seedlings were transferred individually to sterile 10 × 10 cm square Petri dishes with roots draped on a cellophane sheet on top of 10% Modified Melin Norkran’s medium^50^ and inoculated with the ectomycorrhizal fungus *Paxillus involutus*. The shoots of *P. sylvestris* protruded outside the dish through a narrow hole that was sealed around the plant stem with sterile anhydrous lanolin (BDH, Prolabo) to exclude microbial contamination^49^. After 10 weeks, the plants had formed mycorrhizal roots and were transplanted into sterile experimental microcosms that were assembled in a laminar air-flow cabinet. The square Petri dish microcosms contained 100 mL modified Rorison’s^51^ nutrient solution (Supplementary Table S1) beneath a cellophane sheet and was solidified with 1.5% noble agar (Becton Dickinson, Oxford, UK). A single-grain-thickness bed of acid-washed and sieved perlite (volcanic glass) grains of 2.0–2.4 mm was set in a 20 mL layer of noble agar on top of the cellophane (solutions, perlite and cellophane were autoclaved). Six autoclaved plastic wells of 20 mm diameter were inserted as weathering arenas^22^. To these wells, 500 μL of 0.8% noble agar was added and overlaid with a disc of cellophane onto which 0.4 or 0.5 g of sterile mineral/rock samples was placed (one type of rock/mineral per microcosm).

The majority of the microcosms were harvested after 106–125 days of incubation. However, three microcosms with quartz, olivine and granite were harvested after 225–312 days due to late fungal colonization of the wells. All microcosms were watered with sterile water (perlite layer) in 90 day intervals (weathering arenas did not receive any added water). On harvesting, weathering arenas were removed from the microcosms, oven dried (80 °C) and stored in desiccators. Individual hyphae were then removed under 10–50 times magnification using fine forceps, and freed from adhering test mineral grains until all visible hyphae were removed from the wells, leaving the rock/mineral grains behind.

### Quantification of plant-to-fungal carbon allocation to the weathering arenas

After 16–19 weeks of growth, a subset of microcosms (eight microcosms per rock/mineral with four wells each) were sealed in gas tight clear acrylic labelling chambers (675 cm^3^) and exposed to 1.036 MBq of ^14^CO_2_ gas, in an illuminated fume hood (photon flux density of 250 μm m^−2^ s^−1^) for pulse labelling as described earlier^49^. Microcosms were destructively harvested approx. 19 hours after labelling, coincident with the time of greatest ^14^C allocation to the mycorrhizal hyphae determined in earlier trials^52^. The wells with rocks/mineral grains were divided into two equal subsamples and dried at 80 °C overnight. Dry weight measurements were recorded and one half of each well sample was oxidised with a Packard 307 sample oxidiser (Packard Instruments, Meriden, CT) to determine total ^14^C concentration as described previously^49^. The oxidiser operated at >97% recovery rate with <0.08% between sample carry-over. Scintillation counting was carried out on a Tri-Carb 3100 TR Liquid Scintillation Analyser (Packard Instruments). The remaining well samples were in part used for the quantification of oxalate and calcium concentrations.

### Fungal pH measurements

Using fine forceps, individual fungal hyphae were taken from intact mesocosms with live plants and fungi growing in symbiosis. Hyphae were removed from wells containing basalt, granite and limestone grains, from triplicate microcosms, and immediately subjected to confocal laser scanning microscopy (CLSM) to determine pH using the molecular probe SNARF4F (Invitrogen, Carlsbad, CA). CLSM was carried out using a Zeiss Axioscope with Meta 510 detector (Zeiss, Jena, Germany) and an argon multiphoton laser at 488 nm. The Meta 510 detector was set up to detect signals at distinct wavelengths of 544–587 nm and 619–661 nm. A 5 μM solution of the SNARF4F molecular probe was distributed directly onto individual fungal hyphae. At a final concentration of 5 μM the SNARF4F molecular probe emits at around 580 nm in acidic conditions and at around 640 nm in neutral conditions. The ratios of the fluorescence signals were used for pH calibration and calculation as described previously^33^.

### Micro Fourier transform infrared spectroscopy (*μ*FT-IR) of fungal hyphae

Measurements by *μ*FT-IR were performed on a Perkin-Elmer Spotlight imaging system (Perkin-Elmer, Waltham, MA). *μ*FT-IR spectra were collected over the range of 4000-700 cm^−1^ wavenumber at a resolution of 1 cm^−1^, a beam diameter of 6.25 *μ*m and an aperture of 50 × 50 μm in reflective mode on an aluminum block. Background removal from the aluminum block was carried out using the Spectrum Spotlight software (Perkin-Elmer). Each of our test rock/minerals (powdered material), and calcium oxalate monohydrate (powdered, purity 99%, Alpha Aesar, Karlsruhe, Germany) together with hyphae from a monoculture of *P. involutus* grown on cellophane over nutrient agar were scanned for reference purposes. *P. involutus* hyphae were collected from weathering arenas (fresh and dried samples without rock or mineral particles attached) as described above, and *μ*FT-IR spectra obtained from samples derived from at least three different microcosms (of each rock/mineral type), from a total of six wells. Five spectra from different locations were acquired for each well at 100 scans each to increase the representativeness of the spectra per well and subsequently combined to a single spectrum. Scanning areas were selected that contained sufficient biomass to obtain a clear signal without adverse scattering effects.

### Scanning electron microscopy with energy dispersive X-ray spectroscopy (SEM-EDS)

SEM-EDS analysis was carried out on sub samples of *P. involutus* hyphae from the wells in the microcosms analyzed through non-destructive *μ*FT-IR (see above) with a CamScan MKII (Cambridge, UK). Fungal hyphae were prepared on double sided carbon tape and coated with carbon using a Speedivac 12E6/1598 carbon coater (Crawley, UK). Backscattering images were recorded to find elements with a high atomic number at an acceleration voltage of 15 kV. Selected crystalline structures were investigated for calcium content using EDS.

### Quantification of calcium and oxalic acid via inductively coupled plasma mass spectrometry (ICP-MS) and ion chromatography

Quantification of calcium and oxalic acid was carried out on handpicked samples of *P. involutus* hyphae with attached secondary minerals. From triplicate microcosms with quartz, granite, olivine, basalt, limestone and gabbro hyphae were taken (microcline was sampled in duplicate due to the loss of a microcosm). *P. involutus* hyphae were weighed to a precision of 10^−6^ g using a Mettler Toledo MT5 micro balance (Mettler-Toledo Ltd., Greifensee, Switzerland) in acid-washed glass HPLC vials (1.0 mL). Samples were boiled for 30 minutes in 0.5 M sulfuric acid in volumes of 0.3 mL and the aqueous phase was subsequently divided: (i) 0.1 mL was subjected to direct ICP-MS analysis (after a 20 times dilution on an Agilent 7500CX, Agilent Technologies; limit of detection 3 μg calcium L^−1^) and (ii) the remnant 0.2 mL was used for oxalic acid extraction.

The quantification of calcium in the hyphal samples was carried out by measuring Ca^44^ via ICP-MS (Agilent 7500cx, Santa Clara, CA). Isotope fractionation by the fungus was assumed to be negligible since previous studies reported isotopic discrimination of calcium of below 1 ppm in ectomycorrhizal trees^53^.

Oxalic acid quantification was adapted from Lapeyrie and colleagues^19^. Briefly, 0.2 mL of the aqueous phase (see above) was mixed with 0.3 mL of organic solvent tributylphosphate (Acros Organics, Antwerpen, Belgium) in 1.5 mL reaction tubes for 10 min at room temperature on an overhead shaker (RM-2, Elmi, Riga, Latvia) at 20 rpm. The organic phase containing the putative oxalate from the extraction was then pipetted to a new reaction tube and was mixed with 0.2 mL of 2 M NaOH and centrifuged at 16000 *g* for 10 min. 160 μL of the aqueous phase (with the putative oxalate) was neutralized with 160 μL 2 M HCl. The concentration of oxalate was quantified with a ICS1500 ion chromatography system using a 10 μL injection loop, an AS auto sampler, an AS23 column with AG23 guard (all Dionex, Sunnyvale, CA) and a sodium (bi)carbonate based eluent^54^.

Absolute quantifications of calcium and oxalate were calculated per gram dry weight of the fungal biomass including the mass of attached secondary minerals. Additionally, based on the observed timescale of weathering arena colonization (last time point of arenas without fungal colonization to harvest, based on photographic records), and the conversion of fungal biomass into hyphal length (8.64 × 10^4^ m g^−1^)^55^ it was possible to calculate calcium and oxalate accumulation rates per metre of hyphae, per day.

### Calcium accumulation rate calculation

Rate calculations were based on (i) the quantification of calcium within and attached to the sampled fungal hyphae (see above), ii) the fungal biomass present in the weathering arenas at the time of harvesting, and iii) the duration of fungal colonisation of the weathering arenas. The measured fungal biomass was used to calculate a weathering arena specific, maximum possible fungal-mineral contact area (***A***, m^2^), following (Eq. 1):


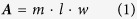


where *m* is the measured mass of fungi in the weathering arena (g), *l* represents the length of hyphae per gram of biomass (8.64 × 10^4^ m g^−1^)^55^, and *w* an assumed hyphal width from previous observations (5 × 10^−6^ m)^35^,^56^. Fungal calcium (Ca) accumulation rates (*R*, moles_Ca_ m^−2^ s^−1^), driven by fungal mineral weathering, were calculated as follows (Eq. 2):


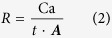


where Ca is the number of moles of calcium measured by ICP-MS, *t* represents the time, in seconds, of the colonization of the wells, and ***A*** the maximum hypha-mineral contact area.

Published chemical dissolution rate laws for minerals in aqueous laboratory solutions are based on 298 K^26^,^28^. In contrast, dissolution *in vivo* for temperate forests takes place under much cooler conditions. In this study, microcosms were incubated with 8 h night at 283 K and 16 h day at 288 K. Therefore, the published abiotic dissolution rates presented in this study were corrected from 298 K to 288 K using the Arrhenius equation as described previously^57^. Abiotic dissolution rates for individual minerals were, where needed, corrected for rate of calcium release from the stoichiometric dissolution rates reported.

### Statistical analysis

Analyis of variance (ANOVA) was tested for all analysed data using Tukey HSD post hoc test where the data meet the requirements of homoscedasticity, as confirmed by Leven’s test. When the raw data showed distributions that were not not normally distributed they were transformed by taking the square root or the log to the base 10. Where the Levene’s test indicated heteroscedasticity in both the raw and transformed data, the Games-Howell posthoc test was used, provided a Welch test indicated significant differences between the sample means. When several weathering arenas were analysed from a single microcosm, the values were used to create means to exclude any non-independent artefacts in the statistical analysis. Error ranges in all tables are based on standard deviation.

## Additional Information

**How to cite this article**: Schmalenberger, A. *et al*. Oxalate secretion by ectomycorrhizal *Paxillus involutus* is mineral-specific and controls calcium weathering from minerals. *Sci. Rep*. **5**, 12187; doi: 10.1038/srep12187 (2015).

## Supplementary Material

Supplementary Information

## Figures and Tables

**Table 1:** Major elements (% wt.) are expressed as oxides of silicon (SiO_2_), aluminium (Al_2_O_3_), magnesium (MgO), potassium (K_2_O), sodium (Na_2_O), calcium (CaO), iron (Fe_2_O_3_) and phosphorus (P_2_O_5_).

	SiO_2_	Al_2_O_3_	CaO	MgO	K_2_O	Na_2_O	Total base cation oxides	Fe_2_O_3_	P_2_O_5_
Quartz	99.90	0.05	0.01	nd	0.01	nd	0.02	0.02	0.006
Microcline	63.97	18.66	0.06	0.02	15.17	0.94	16.18	0.11	0.092
Olivine	43.77	0.90	0.17	45.12	0.01	0.11	45.41	8.99	0.004
Perlite*	75.94	12.75	0.90	0.16	3.28	3.61	7.95	0.83	0.047
Granite	64.88	16.39	3.58	2.26	4.19	3.23	13.25	4.10	0.361
Basalt	46.58	18.39	10.93	8.17	0.30	2.12	21.51	11.63	0.246
Gabbro	46.49	19.64	14.94	10.42	0.08	1.44	26.87	6.10	0.025
Limestone	16.05	2.07	31.75	13.88	0.32	0.30	46.25	1.48	0.111

Values of 0.001 to 0.01 incur an error rate of better than 10% wt; nd = values below 0.001 wt %. *Perlite was added to the main rooting compartment of all mesocosms, but was not present in the wells to which the other rocks and minerals listed here were added.

**Table 2:**
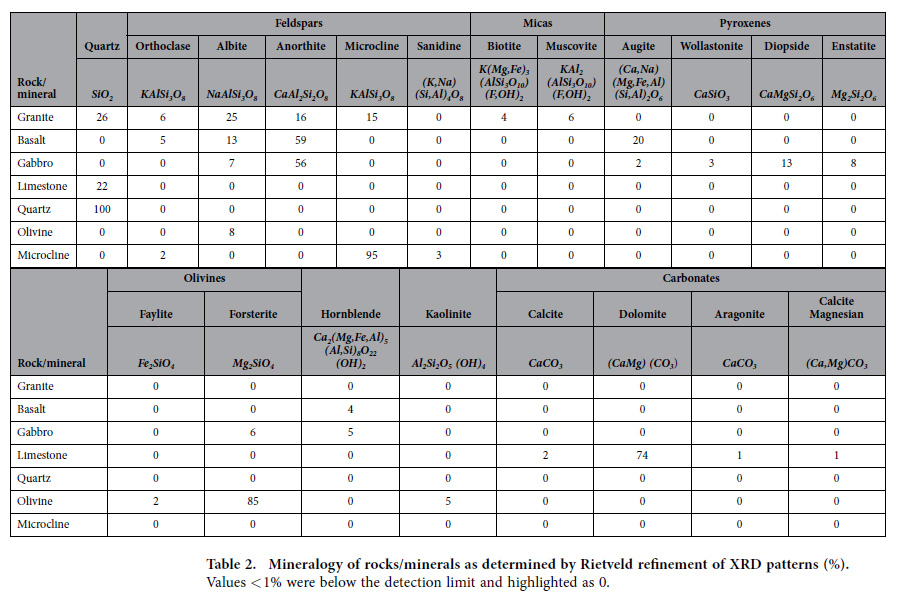
Mineralogy of rocks/minerals as determined by Rietveld refinement of XRD patterns (%).

**Table 3:** Accumulation of oxalate and calcium (mg g^−1^) biomass (DW) on *P. involutus* hyphae (incl. secondary minerals).

Weathering arena Rock/mineral	Oxalate		Calcium	
	mean	±	mean	±
Quartz	3.08^a^	3.17	2.69^a^	2.39
Granite	5.58^a^	5.76	3.07^a^	2.72
Microcline	35.04	7.33	22.37	2.84
Olivine	72.36^b^	19.21	32.99^ab^	17.75
Basalt	63.75^b^	32.4	43.64^b^	18.66
Limestone	77.30^b^	4.58	57.15^b^	8.28
Gabbro	197.91^c^	20.77	112.73^c^	11.53

Letters (a, b, c) indicate significant differences (ANOVA, Tukey); ± indicates standard deviation.

**Table 4:** Oxalate and calcium accumulation rates (10^−10^ mol) per m hyphae and day (m^−1^ d^−1^).

Weathering arena rock/mineral	Oxalate		Calcium	
	mean	±	mean	±
Quartz	0.033^a^	0.035	0.065^a^	0.613
Granite	0.034^a^	0.030	0.047^a^	0.047
Microcline	0.383	0.053	0.551	0.675
Olivine	0.610^b^	0.159	0.591^ab^	0.718
Basalt	0.732^b^	0.521	1.149^ab^	0.569
Limestone	0.845^b^	0.367	1.395^ab^	0.414
Gabbro	2.184^c^	0.250	2.795^b^	0.485

Letters (a, b, c) indicate significant differences (ANOVA; Tukey for oxalate and Games-Howell for calcium data); ± indicates standard deviation.

**Table 5:** (A) Calcium accumulation rates based on theoretical maximum fungal-rock contact area^ł^. (B) Chemical weathering at 288 K in water* [mol m^−2^ s^−1^].

(A)				(B)		
Rock/mineral	Major calcium bearing minerals [>1 mol%]	Calcium accumulation [mol m^−2^ s^−1^]			pH4	pH7
		*R*_1_	±			
Granite	Anorthite	3.2 × 10^−11^	3.2 × 10^−12^	Anorthite	2 × 10^−11^	6 × 10^−12^
Basalt	Anorthite, augite, hornblende	5.6 × 10^−10^	2.7 × 10^−10^	Diopside	3 × 10^−11^	1 × 10^−13^
Limestone	Dolomite	6.6 × 10^−10^	5.6 × 10^−11^	Augite	2 × 10^−12^	>10^−14^
Gabbro	Anorthite, diopside, augite, hornblende	1.1 × 10^−9^	5.7 × 10^−10^	Hornblende	1 × 10^−12^	2 × 10^−13^
				Dolomite	1 × 10^−6^	

^ł^contact area as calculated in methods.

± indicates standard deviation.

^*^modified (Arrhenius correction to 288 K) from^26^,^28^,^38^.

## References

[b1] Anderson I. C. & Cairney J. W. G. Diversity and ecology of soil fungal communities: Increased understanding through the application of molecular techniques. Environ. Microbiol. 6, 769–779 (2004). 15250879 10.1111/j.1462-2920.2004.00675.x

[b2] Brantley S. L. . Twelve testable hypotheses on the geobiology of weathering. Geobiology 9, 140–165 (2011). 21231992 10.1111/j.1472-4669.2010.00264.x

[b3] Turner B. L. . Soil microbial biomass and the fate of phosphorus during long-term ecosystem development. Plant Soil 367, 225–234 (2013).

[b4] Peltzer D. A. . Understanding ecosystem retrogression. Ecol. Monogr. 80, 509–529 (2010).

[b5] Turner B. L., Condron L. M., Wells A. & Andersen K. M. Soil nutrient dynamics during podzol development under lowland temperate rain forest in New Zealand. Catena 97, 50–62 (2012).

[b6] Berner E. K., Berner R. A. & Moulton K. L. in Treatise on Geochemistry (eds Holland H. D. & Turekian K. K. ) Ch. 5.06, 169–188 (Pergamon, 2003).

[b7] Taylor L. L. . Biological weathering and the long-term carbon cycle: integrating mycorrhizal evolution and function into the current paradigm. Geobiology 7, 171–191 (2009). 19323695 10.1111/j.1472-4669.2009.00194.x

[b8] Landeweert R., Hoffland E., Finlay R. D., Kuyper T. W. & Van Breemen N. Linking plants to rocks: Ectomycorrhizal fungi mobilize nutrients from minerals. Trends Ecol. Evol. 16, 248–254 (2001). 11301154 10.1016/s0169-5347(01)02122-x

[b9] Smith S. E. & Read D. J. Mycorrhizal symbiosis. (Academic Press, Amsterdam, The Netherlands, 2008).

[b10] Leake J. R. . Networks of power and influence: the role of mycorrhizal mycelium in controlling plant communities and agroecosystem functioning. Can. J. Bot. 82, 1016–1045 (2004).

[b11] Jones D. L. Organic acids in the rhizosphere - a critical review. Plant Soil 205, 25–44 (1998).

[b12] Parniske M. Arbuscular mycorrhiza: the mother of plant root endosymbioses. Nature Rev. Microbiol. 6, 763–775 (2008). 18794914 10.1038/nrmicro1987

[b13] Read D. J., Leake J. R. & Perez-Moreno J. Mycorrhizal fungi as drivers of ecosystem processes in heathland and boreal forest biomes. Can. J. Bot. 82, 1243–1263 (2004).

[b14] Bonan G. B. Forests and climate change: forcings, feedbacks, and the climate benefits of forests. Science 320, 1444–1449 (2008). 18556546 10.1126/science.1155121

[b15] Klass D. L. in Encyclopedia of Energy (ed Cleveland C. J. ) 193–212 (Elsevier, 2004).

[b16] Hoffland E. . The role of fungi in weathering. Front. Ecol. Environ. 2, 258–264 (2004).

[b17] Leake J. R. . Biological weathering in soil: The role of symbiotic root-associated fungi biosensing minerals and directing photosynthate-energy into grain-scale mineral weathering. Mineral. Mag. 72, 85–89 (2008).

[b18] Quirk J., Andrews M. Y., Leake J. R., Banwart S. A. & Beerling D. J. Ectomycorrhizal fungi and past high CO_2_ atmospheres enhance mineral weathering through increased below-ground carbon-energy fluxes. Biol. Lett. 10 (2014). 10.1098/rsbl.2014.0375 PMC4126629 25115032

[b19] Lapeyrie F., Chilvers G. A. & Bhem C. A. Oxalic acid synthesis by the mycorrhizal fungus *Paxillus involutus* (Batsch EX FR) FR. New Phytol. 106, 139–146 (1987).

[b20] Lapeyrie F. Oxalate synthesis from soil bicarbonate by the mycorrhizal fungus *Paxillus involutus*. Plant Soil 110, 3–8 (1988).

[b21] Lapeyrie F., Ranger J. & Vairelles D. Phosphate-solubilizing activity of ectomycorrhizal fungi *in vitro*. Can. J. Bot. 69, 342–346 (1991).

[b22] Smits M. M., Bonneville S., Benning L. G., Banwart S. A. & Leake J. R. Plant-driven weathering of apatite – the role of an ectomycorrhizal fungus. Geobiology 10, 445–456 (2012). 22624799 10.1111/j.1472-4669.2012.00331.x

[b23] Van Hees P. A. W. . Oxalate and ferricrocin exudation by the extramatrical mycelium of an ectomycorrhizal fungus in symbiosis with *Pinus sylvestris*. New Phytol. 169, 367–378 (2006). 16411939 10.1111/j.1469-8137.2005.01600.x

[b24] Quirk J. . Evolution of trees and mycorrhizal fungi intensifies silicate mineral weathering. Biol. Lett. 8, 1006–1011 (2012). 22859556 10.1098/rsbl.2012.0503 PMC3497110

[b25] Wallander H. Uptake of P from apatite by *Pinus sylvestris* seedlings colonised by different ectomycorrhizal fungi. Plant Soil 218, 249–256 (2000).

[b26] Brantley S. L. in Kinetics of water-rock interaction (eds Brantley S. L., Kubicki J. D. & White A. F. ) 151–210 (Springer, 2008).

[b27] Hausrath E. M., Neaman A. & Brantley S. L. Elemental release rates from dissolving basalt and granite with and without organic ligands. Am. J. Sci. 309, 633–660 (2009).

[b28] McAdam A. C., Zolotov M. Y., Sharp T. G. & Leshin L. A. Preferential low-pH dissolution of pyroxene in plagioclase-pyroxene mixtures: implications for martian surface materials. ICARUS 196, 90–96 (2008).

[b29] Ullman W. J., Kirchman D. L., Welch S. A. & Vandevivere P. Laboratory evidence for microbially mediated silicate mineral dissolution in nature. Chem. Geol. 132, 11–17 (1996).

[b30] Griffiths R. P., Baham J. E. & Caldwell B. A. Soil solution chemistry of ectomycorrhizal mats in forest soil. Soil Biol. Biochem. 26, 331–337 (1994).

[b31] Ilvesniemi H. . Water balance of a boreal Scots pine forest. Boreal Environ. Res. 15, 375–396 (2010).

[b32] Gabler R. E., Petersen J. F. & Trapasso L. M. Essentials of Physical Geography. 8 edn, (Thompson Higher Education, 2006).

[b33] Bonneville S. . Tree-mycorrhiza symbiosis accelerate mineral weathering: evidences from nanometer-scale elemental fluxes at the hypha-mineral interface. Geochim. Cosmochim. Ac. 75, 6988–7005 (2011).

[b34] Bonneville S. . Plant-driven fungal weathering: Early stages of mineral alteration at the nanometer scale. Geology 37, 615–618 (2009).

[b35] Saccone L. . High resolution characterization of ectomycorrhizal fungal-mineral interactions in axenic microcosm experiments. Biogeochemistry 111, 411–425 (2012).

[b36] Hunt J. M., Wisherd M. P. & Bonham L. C. Infrared absorption spectra of minerals and other inorganic compounds. Anal. Chem. 22, 1478–1497 (1950).

[b37] Naumann A. A novel procedure for strain classification of fungal mycelium by cluster and artificial neural network analysis of Fourier transform infrared (FTIR) spectra. Analyst 134, 1215–1223 (2009). 19475151 10.1039/b821286d

[b38] Gautelier M., Oelkers E. H. & Schott J. An experimental study of dolomite dissolution rates as a function of pH from −0.5 to 5 and temperature from 25 to 80°C. Chem. Geol. 157, 13–26 (1999).

[b39] Tewari J. P., Shinners T. C. & Briggs K. G. Production of calcium oxalate crystals by two species of *Cyathus* in culture and infested plant debris. Z. Naturforsch. C-a J. Biosci. 52, 421–425 (1997).

[b40] Maurice-Estepa L., Levillain P., Lacour B. & Daudon M. Advantage of zero-crossing-point first-derivative spectrophotometry for the quantification of calcium oxalate crystalline phases by infrared spectrophotometry. Clin. Chim. Acta 298, 1–11 (2000). 10876000 10.1016/s0009-8981(00)00224-2

[b41] Marschner H. Mineral nutrition of higher plants. 2nd edn, (Academic Press, 1995).

[b42] Jahnke R. A. Vol. 50 Global Biogeochemical Cycles (eds Butcher S. S., Charlson R. J., Orians G. H. & Wolfe G. V. ) 301–315 (Elsevier, 1992).

[b43] Jarosz-Wilkolazka A. & Gadd G. M. Oxalate production by wood-rotting fungi growing in toxic metal-amended medium. Chemosphere 52, 541–547 (2003). 12738291 10.1016/S0045-6535(03)00235-2

[b44] Wallander H., Johansson L. & Pallon J. PIXE analysis to estimate the elemental composition of ectomycorrhizal rhizomorphs grown in contact with different minerals in forest soil. FEMS Microbiol. Ecol. 39, 147–156 (2002). 19709194 10.1111/j.1574-6941.2002.tb00916.x

[b45] Sahin N. Oxalotrophic bacteria. Res. Microbiol. 154, 399–407 (2003). 12892846 10.1016/S0923-2508(03)00112-8

[b46] Taylor L. L., Banwart S. A., Leake J. R. & Beerling D. J. Modeling the evolutionary rise of ectomycorrhiza on sub-surface weathering environments and the geochemical carbon cycle. Am. J. Sci. 311, 369–403 (2011).

[b47] Taylor L. L., Banwart S. A., Valdes P. J., Leake J. R. & Beerling D. J. Evaluating the effects of terrestrial ecosystems, climate and carbon dioxide on weathering over geological time: a global-scale process-based approach. Philos. T. R. Soc. B 367, 565–582 (2012). 10.1098/rstb.2011.0251 PMC3248708 22232768

[b48] Quirk J., Leake J. R., Banwart S. A., Taylor L. L. & Beerling D. J. Weathering by tree-root-associating fungi diminishes under simulated Cenozoic atmospheric CO_2_ decline. Biogeosciences 11, 321–331 (2014).

[b49] Leake J. R., Donnelly D. P., Saunders E. M., Boddy L. & Read D. J. Rates and quantities of carbon flux to ectomycorrhizal mycelium following ^14^C pulse labeling of *Pinus sylvestris* seedlings: effects of litter patches and interaction a wood-decomposer fungus. Tree Physiol. 21, 71–82 (2001). 11303651 10.1093/treephys/21.2-3.71

[b50] Marx D. H. Influence of ectotrophic mycorrhizal fungi on resistance of pine roots to pathogenic infections. I. Antagonism of mycorrhizal fungi to root pathogenic fungi and soil bacteria. Phytopathology 59, 153–163 (1969). 5811914

[b51] Hewitt E. J. Sand and Water Culture Methods used in the Study of Plant Nutrition. 2nd edn, (Commonwealth Agric. Bureaux Tech. Comm. No. 22, 1966).

[b52] Duran A. L. The effect of mineral particle grain size and mineralogy on ectomycorrhizal-mineral interactions PhD thesis, Univeristy of Sheffield, (2014).

[b53] Hoff C. J., Bryce J. G., Hobbie E. A., Colpaert J. V. & Bullen T. D. in *American Geophysical Union, Fall Meeting*. B53B-05.

[b54] Schmalenberger A., Telford A. & Kertesz M. A. Sulfate treatment affects desulfonating bacterial community structures in *Agrostis* rhizospheres as revealed by functional gene analysis based on *asfA*. Eur. J. Soil Biol. 46, 248–254 (2010).

[b55] Wallander H., Göransson H. & Rosengren U. Production, standing biomass and natural abundance of ^15^N and ^13^C in ectomycorrhizal mycelia collected at different soil depths in two forest types. Oecologia 139, 89–97 (2004). 14727173 10.1007/s00442-003-1477-z

[b56] Gazzè S. A. . Nanoscale observations of extracellular polymeric substances deposition on phyllosilicates by an ectomycorrhizal fungus. Geomicrobiol. J. 30, 721–730 (2013).

[b57] Rosling A., Lindahl B. D., Taylor A. F. S. & Finlay R. D. Mycelial growth and substrate acidification of ectomycorrhizal fungi in response to different minerals. FEMS Microbiol. Ecol. 47, 31–37 (2004). 19712344 10.1016/S0168-6496(03)00222-8

