# Supplementary material for: Oxalate secretion by ectomycorrhizal Paxillus involutus is mineral-specific and controls calcium weathering from minerals
Source: Sci Rep. 2015 Jul 22;5:12187. doi: 10.1038/srep12187 (PMC4510491; doi:10.1038/srep12187)
Supplement: Supplementary Information [file srep12187-s1.doc]

Supplementary material

Oxalate secretion by ectomycorrhizal *Paxillus involutus* is mineral-specific and controls calcium weathering from minerals

*A. Schmalenberger^1,2,3^, A.L. Duran^2^, A.W. Bray^4^, J. Bridge^1^, S. Bonneville^4ǂ^, L.G. Benning^4^, M.E. Romero-Gonzalez^1^, J.R. Leake^2^ and S.A. Banwart^1*^*

Supplementary Figure S1

Scanning Electron Microscopy images in backscattering mode from symbiotic hyphae of *P. involutus* taken from weathering arenas with granite (A), basalt (B) and limestone (C). Pixels with high reflexion represent elements of high atomic number (identified as calcium via EDS).


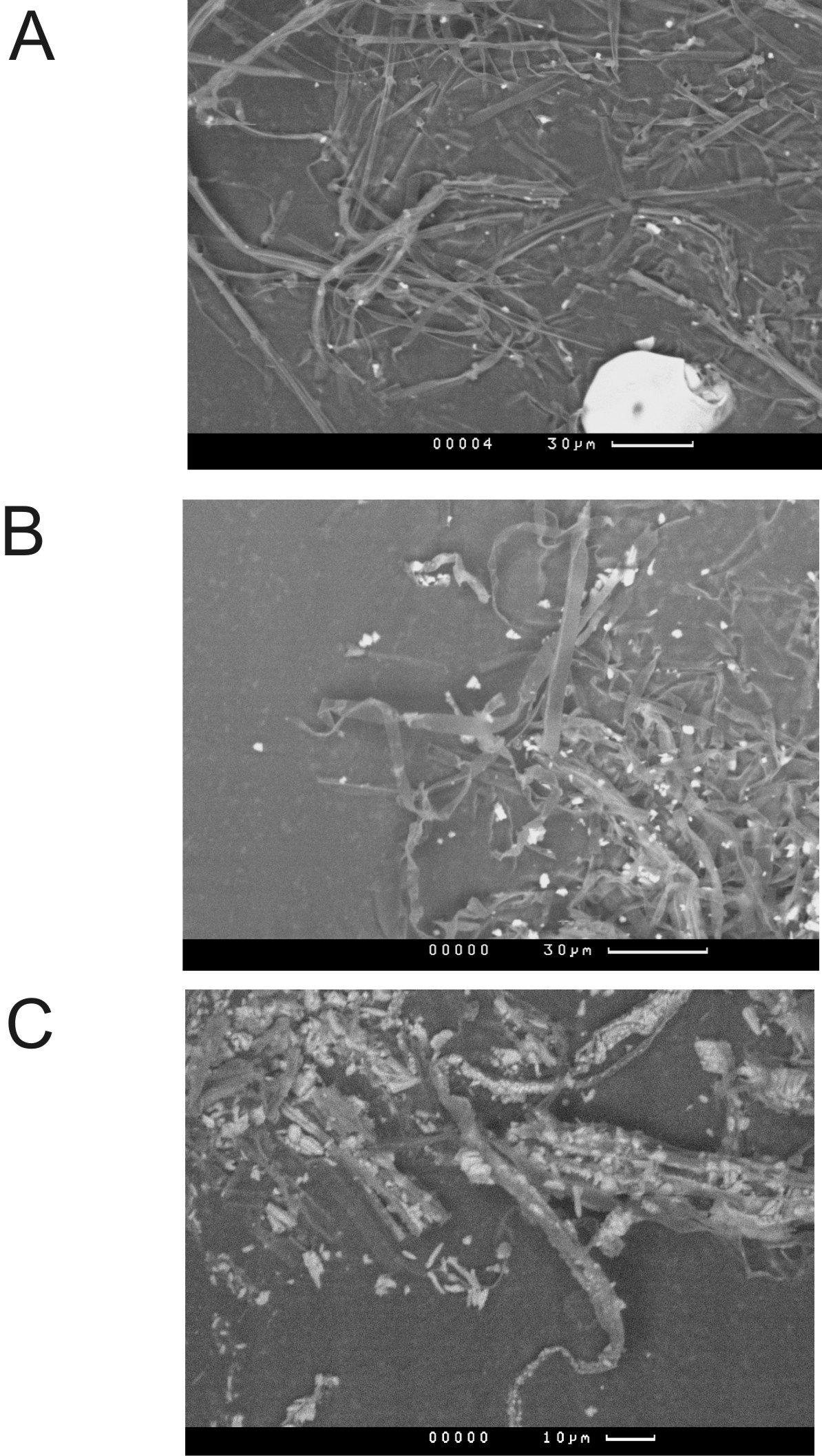


Supplementary Figure S2

Correlation of carbon (^14^C) and calcium accumulation in weathering arenas (DPM or mg g^-1^ DW) with quartz (empty diamond), granite (empty square), microcline (empty triangle), olivine (empty circle), basalt (filled square) and gabbro (filled circle). R-square value of linear regression fit is 0.0.688 (black line).


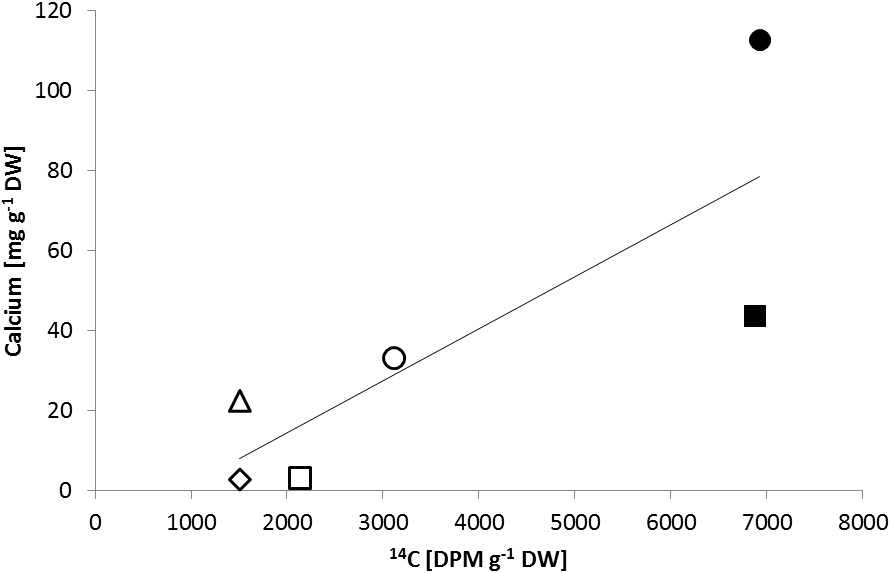


Supplementary Table S1

S1a - Nutrient content of bottom layer of agar in the microcosms

|  | mg l^-1^ |
| --- | --- |
| Ammonium sulfate | 229.1 |
| Magnesium sulfate heptahydrate | 248 |
| Iron EDTA | 25 |
| Manganese sulfate tetrahydrate | 2.028 |
| Boric acid | 2.865 |
| Ammonium molybdate tetrahydrate | 0.184 |
| Zinc sulfate heptahydrate | 0.44 |
| Copper sulfate pentahydrate | 0.393 |
| Calcium nitrate tetrahydrate | 17.7 |

S1b - Nutrient content of top layer of agar in the microcosms, anchoring perlite grains

|  | mg l^-1^ |
| --- | --- |
| Ammonium sulfate | 229.1 |
| Magnesium sulfate heptahydrate | 248 |
| Iron EDTA | 25 |
| Manganese sulfate tetrahydrate | 2.028 |
| Boric acid | 2.865 |
| Ammomium molybdate tetrahydrate | 0.184 |
| Zinc sulfate heptahydrate | 0.44 |
| Copper sulfate pentahydrate | 0.393 |
| Calcium nitrate tetrahydrate | 17.7 |
| Di Ammonium hydrogen orthophosphate | 76.75 |

Potassium is indirectly supplied either through the perlite grains in the main compartment or the rocks/minerals in wells in the microcosms.
